# Epidural anesthesia and postoperative analgesia with ropivacaine and fentanyl in off-pump coronary artery bypass grafting: a randomized, controlled study

**DOI:** 10.1186/1471-2253-11-17

**Published:** 2011-09-18

**Authors:** Mikhail Y Kirov, Alexey V Eremeev, Alexey A Smetkin, Lars J Bjertnaes

**Affiliations:** 1 Department of Anesthesiology and Intensive Care Medicine, Northern State Medical University, Troitsky avenue 51, Arkhangelsk, 163000, Russian Federation; 2 Department of Anesthesiology and Intensive Care Medicine, City Hospital #1, Suvorov Street 1, Arkhangelsk, 163001, Russian Federation; 3 Department of Clinical Medicine (Anesthesiology), Faculty of Medicine, University of Tromsø, MH-Breivika, Tromsø, 9038, Norway; 4 Department of Anesthesiology, University Hospital of North Norway, Sykehusveien 38, Tromsø, 9038, Norway

**Keywords:** epidural anesthesia, analgesia, patient-controlled analgesia, off-pump coronary artery bypass grafting

## Abstract

**Background:**

Our aim was to assess the efficacy of thoracic epidural anesthesia (EA) followed by postoperative epidural infusion (EI) and patient-controlled epidural analgesia (PCEA) with ropivacaine/fentanyl in off-pump coronary artery bypass grafting (OPCAB).

**Methods:**

In a prospective study, 93 patients were scheduled for OPCAB under propofol/fentanyl anesthesia and randomized to three postoperative analgesia regimens aiming at a visual analog scale (VAS) score < 30 mm at rest. The control group (n = 31) received intravenous fentanyl 10 μg/ml postoperatively 3-8 mL/h. After placement of an epidural catheter at the level of Th_2_-Th_4 _before OPCAB, a thoracic EI group (n = 31) received EA intraoperatively with ropivacaine 0.75% 1 mg/kg and fentanyl 1 μg/kg followed by continuous EI of ropivacaine 0.2% 3-8 mL/h and fentanyl 2 μg/mL postoperatively. The PCEA group (n = 31), in addition to EA and EI, received PCEA (ropivacaine/fentanyl bolus 1 mL, lock-out interval 12 min) postoperatively. Hemodynamics and blood gases were measured throughout 24 h after OPCAB.

**Results:**

During OPCAB, EA decreased arterial pressure transiently, counteracted changes in global ejection fraction and accumulation of extravascular lung water, and reduced the consumption of propofol by 15%, fentanyl by 50% and nitroglycerin by a 7-fold, but increased the requirements in colloids and vasopressors by 2- and 3-fold, respectively (*P *< 0.05). After OPCAB, PCEA increased PaO_2_/FiO_2 _at 18 h and decreased the duration of mechanical ventilation by 32% compared with the control group (*P *< 0.05).

**Conclusions:**

In OPCAB, EA with ropivacaine/fentanyl decreases arterial pressure transiently, optimizes myocardial performance and influences the perioperative fluid and vasoactive therapy. Postoperative EI combined with PCEA improves lung function and reduces time to extubation.

**Trial Registration:**

NCT01384175

## Background

Coronary artery bypass grafting (CABG) is one of the most common cardiosurgical interventions. In many institutions, CABG is performed without cardiopulmonary bypass (CPB), a modification which is commonly referred to as off-pump coronary artery bypass grafting (OPCAB) [1-4]. The off-pump technique enables coronary revascularization on the beating heart, thereby reducing the risk of complications associated with CPB. However, OPCAB can be accompanied by hemodynamic alterations, postoperative pain, and respiratory dysfunction, requiring thorough monitoring and perioperative care [3-6].

In cardiosurgical patients, high thoracic epidural anesthesia (EA) with local anesthetics and opioids can provide effective analgesia and reduce the number of perioperative complications [6-8]. However, the use of EA in coronary surgery is controversial, and it is still unclear whether EA influences lung fluid balance, cardiopulmonary function and clinical outcome in OPCAB. Thus, the method requires further evaluation and its potential benefits in coronary patients should be weighed against its risks [8,9].

Patient-controlled epidural analgesia (PCEA) is an attractive technique for postoperative pain relief. However, in OPCAB the role of PCEA with administration of a ropivacaine/fentanyl mixture via a thoracic epidural catheter remains unsettled.

We hypothesized that after OPCAB, thoracic epidural analgesia with ropivacaine and fentanyl aiming at a visual analog scale (VAS) score < 30 mm will be associated with improved cardiopulmonary parameters in comparison with intravenously administered analgesia. If the hypothesis is confirmed, we expect that epidural administration of ropivacaine and fentanyl, including a patient-controlled mode, might reduce the duration of mechanical ventilation. Thus, the duration of mechanical ventilation and the changes in cardiopulmonary variables served as the primary and the secondary study end-points, respectively. Using these end-points, the aim of our study was to assess the influence of EA followed by postoperative PCEA with ropivacaine/fentanyl on cardiopulmonary function in the perioperative management of OPCAB patients.

## Methods

### Patients

The study protocol and informed consent form were approved by the Ethics Committee of Northern State Medical University, Arkhangelsk, Russian Federation. Written informed consent was obtained from every patient.

The study was performed in a 900-bed university hospital. During the period from January of 2008 to September of 2009, 93 adult patients with coronary artery disease, ranked ASA III and scheduled for elective OPCAB, were enrolled into a prospective randomized study. Exclusion criteria were age < 18 years, severe valve dysfunction or peripheral vascular disease, simultaneous interventions (carotid endarterectomy, aneurysm repair, *etc*.), or transfer to CPB during surgery.

### Anesthesia and surgery

All patients received standard premedication with diazepam (Seduxen; Gedeon Richter, Budapest, Hungary). After establishment of routine hemodynamic monitoring with ECG including ST segment analysis, blood pressure, and SpO_2 _measurements, anesthesia was induced with intravenous midazolam (Dormicum, F. Hoffmann-La Roche Ltd., Switzerland) 0.07 mg/kg, propofol (Diprivan, AstraZeneca, UK) 1 mg/kg, and fentanyl (Fentanyl, Moscow Endocrine Factory, Russian Federation) 3-5 μg/kg. Neuromuscular blockade was induced with intravenous pipecuronium (Arduan, Gedeon Richter, Hungary) 0.1 mg/kg and maintained with repeated doses of pipecuronium 0.015 mg/kg/h. Anesthesia was maintained with propofol 3 mg/kg/h from the onset and continuing with 3-5 mg/kg/h, and fentanyl 2-4 μg/kg/h, aiming at heart rate within 50-90 beats/min and mean arterial pressure within 60-80 mm Hg.

Mechanical ventilation in the operating room was performed by using a semi-closed anesthetic circuit (Fabius, Dräger, Germany) with FiO_2 _0.5, tidal volume 7-8 mL/kg, respiratory rate 12-14/min aiming at a PaCO_2 _of 35-45 mm Hg, positive end-expiratory pressure 4 cm H_2_O, and fresh gas flow of 1 L/min.

**Figure 1:**
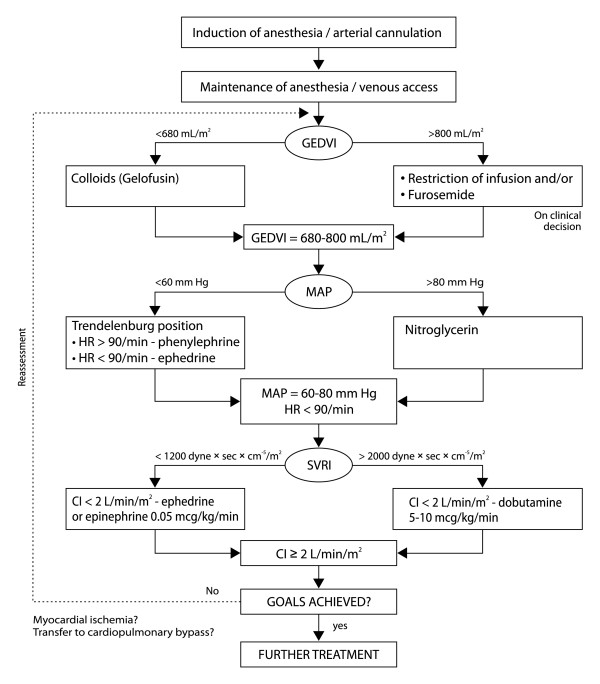
**Flowchart showing algorithm for hemodynamic monitoring and perioperative management**. MAP: mean arterial pressure; HR: heart rate; GEDVI: global end-diastolic volume index; CI: cardiac index; SVRI: systemic vascular resistance index. During anesthesia, this algorithm was used to correct HR and MAP only if the doses of propofol 3-5 mg/kg/h and fentanyl 2-4 μg/kg/h were unable to maintain HR within 50-90 beats/min and MAP within 60-80 mm Hg. For perioperative management, we used the following doses of intravenous agents: ephedrine 5-10 mg, phenylephrine 0, 05-1 mg, nitroglycerine 0, 3-3 mg/h and furosemide 10-20 mg.

Fluid therapy included infusion of Ringer's lactate at rates of 6-7 mL/kg/h prior to and during anesthesia and 2-3 mL/kg/h during the first 6 h postoperatively. In cases of hypovolemia, diagnosed by volumetric hemodynamic monitoring (Figure 1), we administered Gelofusin (Braun, Germany) 6-8 mL/kg over 30 min. A transfusion trigger was hemoglobin level < 8 g/dL.

All patients were operated by the same team of surgeons using midline sternotomy, and Acrobat SUV OM-9000S (Guidant, Santa Clara, USA) device for stabilization of the heart during revascularization. After pericardiotomy, we administered heparin 100 Units/kg aiming at maintenance of activated clotting time > 250 sec during revascularization.

### Randomization and protocol

The patients were randomized to three groups using the envelope method. All groups received lornoxicam (Xefocam, Nycomed, Austria) 8 mg IV before OPCAB and every 12 h during two postoperative days.

**Figure 2:**
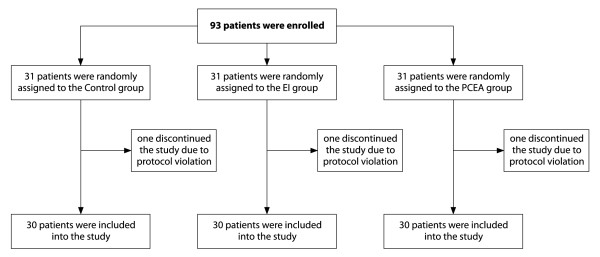
**Flowchart of the study groups**. EI: epidural infusion group; PCEA: patient-controlled epidural analgesia group.

Figure 2 displays a flowchart of the study groups. The control group (n = 31) had no epidural catheter and received fentanyl 10 μg/ml intravenously at a rate of 3-8 mL/h postoperatively.

In two other groups, an epidural catheter (Perifix 16G, Braun, Germany) was inserted at Th_2_-Th_4 _level before induction of anesthesia. The epidural space was identified by the loss-of-resistance technique; 3 to 4 cm of the catheter was inserted into the epidural space followed by administration of a test dose of 1 mL of 2% lidocaine.

After placement of the epidural catheter and induction of anesthesia, the thoracic epidural infusion (EI) group (n = 31) received EA with ropivacaine (Naropin, AstraZeneca, UK) 0.75% 1 mg/kg and fentanyl 1 μg/kg followed by postoperative continuous EI of ropivacaine 0.2% and fentanyl 2 μg/mL at a rate of 3-8 mL/h.

The PCEA group (n = 31) received the same intraoperative therapy as the EI group but, at variance, this was combined with postoperative PCEA using a programmable infusion pump (Graseby 3300, UK) with ropivacaine/fentanyl bolus 1 mL, which could be administered on the patients discretion with a lock-out interval of 12 min. The patients started to use PCEA after recovery from anesthesia.

The postoperative analgesia was administered by the attending physician, who adjusted the infusion rate of fentanyl in the control group and ropivacaine/fentanyl in the EI groups beginning from 3 mL/h and aiming at a VAS score of < 30 mm at rest. All patients received aspirin postoperatively at a daily dose of 75 mg *per os*.

### Measurements

The femoral artery was catheterized with a 5F arterial thermodilution catheter (Pulsiocath PV2015L20, Pulsion, Germany), which was connected to a LifeScope (Nihon Kohden, Japan) and a PiCCO*plus *(Pulsion Medical Systems, Germany) monitor for thermodilution measurements and continuous hemodynamic monitoring. After induction of anesthesia, an 8.5F central venous catheter was inserted into the internal jugular vein. The registered hemodynamic parameters included heart rate (HR), mean arterial pressure (MAP), and central venous pressure (CVP), as well as global end-diastolic volume index (GEDVI), cardiac index (CI), extravascular lung water index (EVLWI), cardiac function index (CFI), global ejection fraction (GEF), left ventricle contractility index (dPmax), and systemic vascular resistance index (SVRI).

The hemodynamic parameters were registered after induction of anesthesia, at the restraint of the heart by using the stabilizing devices, at 5 min after restoration of blood flow *via *the coronary grafts, at the end of surgery, and at 2, 6, 12, 18, and 24 h postoperatively. These time-points were selected for goal-directed hemodynamic adjustments (Figure 1).

Blood gases and plasma lactate concentration were measured after induction of anesthesia, at the end of surgery, and at 2, 6, 12, 18, and 24 h postoperatively using ABL800Flex (Radiometer, Denmark). Plasma concentrations of cortisol and troponin-T were measured before surgery and at 6 and 18 h postoperatively (Roche Diagnostics GmbH, Germany).

The severity of postoperative pain at rest and during coughing was assessed by the attending physician with VAS score at 6, 12, 18, and 24 h after OPCAB. The level of sedation was also evaluated by the attending physician by means of the Ramsay's score. In addition, we recorded the drug consumption, the perioperative fluid balance and administration of fluids, inotropes and vasoactive drugs, perioperative adverse effects, duration of surgery and postoperative mechanical ventilation, lengths of postoperative ICU and hospital stay, and the mortality rate at Day 28.

Extubation criteria were the following: a cooperative, alert patient; adequate muscular tone; SpO_2 _> 95% with FiO_2 _0.5; PaCO_2 _< 45 mm Hg; stable hemodynamics without inotrope/vasopressor support; absence of arrhythmias; and body temperature > 35°C. Temporary pacing was not regarded as a contraindication to extubation.

The patients were discharged from hospital if they satisfied the following criteria: hemodynamic stability, independence in ambulation and feeding, afebrile with clean wound and no obvious infections, normal voiding and bowel movements, pain control on oral medications, and exercise tolerance. The doctor responsible for discharge from the hospital was unaware of the study groups.

### Statistical analysis

For data collection and analysis, we used SPSS software (version 14.0; SPSS Inc., Chicago, IL, USA). Calculation of sample size was based on initial observations (10 cases in each group) and the hypothesis that postoperative PCEA will shorten the time to tracheal extubation by 90 min compared with the control group. In order to find a statistically significant difference with α of 0.05 and power of 0.8, a sample size of 30 patients in each group proved to be sufficient. The normality of the data distribution was assessed using the Shapiro-Wilk test. In case of normal distribution, we used ANOVA and *post hoc *Dunnet test for comparison among the groups and repeated measures ANOVA followed by test of contrasts for intragroup comparisons. Non-parametrically distributed data were assessed by Friedman test followed by Wilcoxon test, respectively. The quantitative values are expressed as mean ± SD and median (25^th^-75^th ^percentiles) in accordance with data distribution. The discrete data were analyzed by two-sided chi-square test and expressed as patient number or percentage. For all tests, a *P*-value < 0.05 was considered as significant.

## Results

As shown in Table 1, we found no significant differences among the groups concerning demographic data, including co-morbidities and preoperative ejection fraction. Three patients (one patient in each group) who became hemodynamically unstable during CABG were transferred to CPB and excluded from further analysis (Figure 2). One of these patients (belonging to the EI group) required re-operation and died because of postoperative bleeding from the aorta. All the other patients survived to Day 28 and were discharged from hospital. Duration of surgery, as well as of ICU and hospital stays, did not differ among the groups. The duration of mechanical ventilation was reduced by 32% in the PCEA group (*P *= 0.04) and tended to decrease in the EI group (*P *= 0.14) compared with the control group (Table 1).

**Table 1:** Main characteristics of patients undergoing OPCAB

Characteristic	Groups		
	Control (n = 30)	EI (n = 30)	PCEA (n = 30)
Age, yrs	58.6 ± 9.1	54.5 ± 8.2	53.6 ± 7.0
Gender, males/females, %	73/27	90/10	90/10
Preoperative ejection fraction	0.58 ± 0.10	0.58 ± 0.08	0.59 ± 0.09
Duration of surgery, min	174 ± 38	149 ± 36	155 ± 40
Duration of mechanical ventilation, min	358 (266-455)	300 (180-385)	245 (199-345)*
Postoperative ICU stay, h	59 ± 29	62 ± 28	59 ± 25
Duration of postoperative hospital stay, days	16.0 (14.8-20.3)	15.0 (14.0-18.5)	15.0 (14.0-18.3)

Data are presented as mean ± SD or median (25^th^-75^th ^percentiles).

* *P *< 0.05 compared with the control group.

Control, control group; EI, epidural infusion group; PCEA, patient controlled epidural analgesia group.

Table 2 displays changes in hemodynamics. In the groups receiving EA, MAP decreased transiently by 10-15% during OPCAB (*P *< 0.05), but rose postoperatively without intergroup differences. After induction of anesthesia, all groups demonstrated reduced HR, CI, CFI, dPmax and GEF, and increased SVRI as compared to normal values. Perioperatively, HR, CI, and CFI rose in all groups whereas SVRI declined (*P *< 0.05). Compared to baseline, CVP increased significantly at the restraint of the heart and then decreased after OPCAB in all groups (*P *< 0.05). Global end-diastolic volume index did not change significantly. In the EI and the PCEA groups, dPmax increased postoperatively by 70-85% (*P *< 0.05). By contrast, in the control group GEF decreased by 10-15% during OPCAB, and EVLWI rose by 22% during the restraint of the heart (*P *< 0.05).

**Table 2:** Hemodynamic parameters in patients undergoing OPCAB

Characteristic	Group	Intraoperative period				Postoperative period				
		After induction of anesthesia	Fixation of the heart	5 min after start flow	End of operation	2 hours	6 hours	12 hours	18 hours	24 hours
	Control	79.9 ± 13.5	76.9 ± 15.6	74.6 ± 14.3	80.2 ± 10.4	89.9 ± 15.3^+^	87.6 ± 12.7^+^	82.5 ± 14.8^#^	80.1 ± 13.1	80.1 ± 13.1
MAP, mm Hg	EI	72.8 ± 8.37	67.8 ± 13.5^+^	72.6 ± 11.8	74.2 ± 12.6	86.1 ± 17.6^+^	81.2 ± 14.9^+^	72.0 ± 12.4	76.1 ± 12.4	75.6 ± 11.4
	PCEA	79.4 ± 13.6	67.5 ± 12.1^+^	74.7 ± 12.7	72.6 ± 11.4^+^	89.6 ± 14.9^+^	89.4 ± 17.5^+^	83.7 ± 17.3^#^	79.1 ± 17.9	82.3 ± 17.0
	Control	50.0 (42.3-59.8)	59.5 (51.3-64.8)^+^	62.5 (50.5-70.5)^#+^	61.0 (55.3-74.5)^#+^	77.5 (71.0-85.0)^+^	87.5 (77.5-98.0)^+^	87.0 (80.3-101.0)^#+^	82.5 (75.0-96.8)^+^	83.5 (77.0-94.5)^+^
HR, 1/min	EI	52.0 (43.3-59.8)	51.5 (44.3-63.8)	54.5 (48.3-62.8)	56.0 (48.3-66.0)	66.5 (56.8-84.0)^+^	85.0 (75.5-88.8)^+^	79.5 (75.3-92.0)^+^	81.0 (73.0-89.8)^+^	80.0 (74.8-88.8)^+^
	PCEA	54.0 (48.5-64.0)	55.0 (49.0-63.0)	58.0 (54.0-65.0)	61.0 (55.0-66.5)	71.0 (64.0-86.5)^+^	91.0 (82.0-105.0)^+^	89.0 (83.0-94.0)^#+^	86.0 (78.5-91.5)^+^	86.0 (80.5-93.5)^+^
	Control	2.20 ± 0.42	2.43 ± 0.59	2.96 ± 1.23^+^	2.70 ± 0.80	3.27 ± 0.74^+^	3.55 ± 0.83^+^	3.69 ± 0.78^+^	3.39 ± 0.38	3.46 ± 0.42^+^
CI, l/min/m^2^	EI	2.20 ± 0.44	1.97 ± 0.51	2.55 ± 0.33^+^	2.57 ± 0.55^+^	3.12 ± 0.69^+^	3.43 ± 0.59^+^	3.43 ± 0.53^+^	3.34 ± 0.50	3.32 ± 0.44^+^
	PCEA	2.15 ± 0.46	1.89 ± 0.51	2.81 ± 0.75^+^	2.58 ± 0.80	3.27 ± 0.87^+^	3.79 ± 0.63^+^	3.46 ± 0.49^+^	3.08 ± 0.44	3.40 ± 0.46^+^
	Control	3.47 ± 1.18	3.14 ± 1.04	3.88 ± 1.42	3.79 ± 1.24	4.71 ± 1.83^+^	5.01 ± 1.73^+^	5.56 ± 1.92^+^	4.96 ± 1.19^+^	4.98 ± 1.11^+^
CFI, 1/min	EI	3.42 ± 0.77	3.03 ± 0.65^+^	3.69 ± 0.69	3.65 ± 0.87	4.68 ± 0.92^+^	5.25 ± 0.76^+^	5.23 ± 1.22^+^	5.13 ± 1.14^+^	4.95 ± 1.29^+^
	PCEA	3.32 ± 0.68	3.08 ± 0.66	3.97 ± 1.04^+^	3.88 ± 1.16^+^	4.47 ± 1.21^+^	5.33 ± 1.56^+^	4.87 ± 1.22^+^	4.28 ± 1.07^+^	4.30 ± 1.14^+^
	Control	2709 ± 500	2484 ± 773	2256 ± 725	2228 ± 568^+^	2150 ± 637	1967 ± 763^+^	1647 ± 648^+^	1731 ± 378^+^	1790 ± 428^+^
SVRI, dyne'sec'cm^-5'^m^-2^	EI	2458 ± 707	2120 ± 600	1933 ± 287^+^	1953 ± 290	1993 ± 482	2035 ± 491^+^	1677 ± 456^+^	1846 ± 277^+^	1788 ± 329^+^
	PCEA	2912 ± 970	2299 ± 791	2059 ± 892	2112 ± 733^+^	2223 ± 554	2086 ± 405	2123 ± 487^+^	2205 ± 623*^+^	2078 ± 604^+^
	Control	11.3 ± 3.6	14.0 ± 4.0^#+^	11.8 ± 3.7^#^	12.7 ± 3.5^#+^	7.6 ± 3.7^+^	5.9 ± 3.1^+^	5.0 ± 4.0^+^	4.6 ± 4.2^+^	5.8 ± 4.6^+^
CVP, mm Hg	EI	12.5 ± 3.1	16.8 ± 3.2^+^	14.4 ± 2.5^+^	14.9 ± 2.3^+^	8.9 ± 3.3^+^	7.2 ± 4.8^+^	6.3 ± 4.2^+^	5.4 ± 5.3^+^	4.9 ± 3.3^+^
	PCEA	11.4 ± 2.67	15.0 ± 3.8^+^	13.0 ± 3.0^+^	13.4 ± 2.8^+^	8.3 ± 4.3^+^	4.3 ± 3.5^#+^	4.1 ± 3.3^+^	4.6 ± 4.0^+^	5.4 ± 3.8^+^
	Control	689 ± 243	797 ± 227	765 ± 168	711 ± 117	729 ± 169	724 ± 165	695 ± 188	715 ± 199	710 ± 145
GEDVI, ml/m^2^	EI	674 ± 215	661 ± 250	696 ± 153	704 ± 251	680 ± 149	659 ± 106	660 ± 125	661 ± 107	682 ± 115
	PCEA	653 ± 143	646 ± 159	722 ± 116	665 ± 146	683 ± 251	728 ± 139	728 ± 133	720 ± 125	799 ± 148
	Control	697 ± 116	537 ± 169	567 ± 142	565 ± 145	803 ± 412	773 ± 307	770 ± 255	744 ± 412	915 ± 339
dPmax, mm Hg/sec	EI	542 ± 136	479 ± 150	552 ± 141	567 ± 201	803 ± 412^+^	849 ± 288^+^	880 ± 293^+^	1015 ± 421^+^	997 ± 353^+^
	PCEA	561 ± 203	457 ± 159	536 ± 179	578 ± 219	843 ± 263^+^	813 ± 177^+^	909 ± 284^+^	877 ± 243^+^	857 ± 195^+^
	Control	24.6 ± 6.1	21.0 ± 6.6^+^	22.2 ± 7.0	22.0 ± 5.6^+^	22.3 ± 5.8	22.3 ± 4.7	24.0 ± 6.3	22.0 ± 5.4	21.7 ± 5.3
GEF, %	EI	26.2 ± 7.30	23.5 ± 5.4	26.1 ± 6.3	26.0 ± 7.0	26.7 ± 7.7	22.6 ± 5.0	24.2 ± 6.1	24.0 ± 5.7	22.9 ± 5.5
	PCEA	21.8 ± 7.2	21.2 ± 6.1	24.6 ± 6.2^+^	23.8 ± 6.5	23.9 ± 5.1	22.8 ± 5.7	21.6 ± 6.3	19.0 ± 5.1	19.6 ± 4.8
	Control	9.0 (7.0-10.0)	11.0 (8.0-14.0)^+^	9.0 (8.0-11.0)	10.0 (7.0-11.0)	8.0 (5.0-12.0)	8.0 (6.0-11.0)	10.0 (6.0-11.0)	8.0 (5.0-14.0)	9.0 (8.0-13.0)
EVLWI, ml/kg	EI	9.0 (7.0-9.5)	8.5 (6.8-10.5)	9.0 (7.0-10.3)	8.0 (6.8-10.0)	7.5 (6.0-11.8)	6.5 (6.0-10.3)	6.0 (5.8-7.3)^+^	7.00 (6.8-8.8)	7.5 (6.0-9.3)
	PCEA	8.0 (6.3-9.8)	9.5 (7.0-11.0)	8.0 (7.3-11.8)	8.0 (7.0-10.5)	8.0 (7.0-12.8)	6.5 (6.0-8.0)	7.0 (5.3-8.8)	7.5 (6.0-9.8)	7.5 (7.0-10.8)

Data are presented as mean ± SD or median (25^th^-75^th ^percentiles).

* *P *< 0.05 compared with the control group;

^# ^*P *< 0.05 compared with the EI group;

^+ ^*P *< 0.05 compared with baseline (after induction of anesthesia).

Control, control group; EI, epidural infusion group; PCEA, patient controlled epidural analgesia group; MAP, mean arterial pressure; HR, heart rate; CI, cardiac index; CFI, cardiac function index; SVRI, systemic vascular resistance index; CVP, central venous pressure; GEDVI, global end-diastolic volume index; dPmax, left ventricle contractility index; GEF, global ejection fraction; EVLWI, extravascular lung water index.

Table 3 demonstrates blood gases and biochemical variables. In all groups, pH declined intraoperatively and during 6 h postoperatively, but increased significantly at 24 h in the groups receiving epidural analgesia. In parallel, PaCO_2 _decreased from intragroup baseline during 12-24 h in the EI group and during 18-24 h in the PCEA group but without differences with control group where PaCO_2 _also reduced at 24 h. At 18 h, PaO_2_/FiO_2 _was higher in the PCEA group (*P *= 0.03 compared with controls). Plasma concentrations of lactate and glucose rose postoperatively in all groups; however, in the PCEA group lactate fell by 33% compared with the control group (*P *= 0.04) at 18 h. After OPCAB, plasma concentrations of cortisol and troponin T increased without intergroup differences.

**Table 3:** Laboratory parameters in patients undergoing OPCAB

Characteristic	Group	Period						
		After induction of anesthesia	End of Operation	2 hours	6 hours	12 hours	18 hours	24 hours
	Control	7.39 ± 0.04	7.34 ± 0.05^+^	7.30 ± 0.04^+^	7.33 ± 0.03^+^	7.36 ± 0.03^+^	7.39 ± 0.03	7.40 ± 0.06
pH	EI	7.37 ± 0.03	7.34 ± 0.04^+^	7.30 ± 0.06^+^	7.34 ± 0.04^+^	7.38 ± 0.03^+^	7.39 ± 0.03^+^	7.41 ± 0.03^+^
	PCEA	7.38 ± 0.03	7.34 ± 0.05^+^	7.30 ± 0.04^+^	7.35 ± 0.04^+^	7.37 ± 0.04	7.40 ± 0.03	7.40 ± 0.03^+^
	Control	38.2 ± 3.49	39.0 ± 5.56	41.1 ± 5.05	39.0 ± 4.21	38.0 ± 5.10	36.8 ± 4.14	33.7 ± 4.13^+^
PaCO_2_, mm Hg	EI	40.1 ± 4.41	39.4 ± 5.16	42.1 ± 6.90	38.5 ± 4.93	35.7 ± 4.82^+^	36.1 ± 3.61^+^	34.2 ± 2.86^+^
	PCEA	39.2 ± 3.77	39.2 ± 5.36	42.1 ± 4.34	37.8 ± 3.29	36.7 ± 3.97	34.5 ± 3.73^+^	33.9 ± 4.38^+^
	Control	287 ± 100	243 ± 108	275 ± 73	282 ± 80	273 ± 76	272 ± 139	302 ± 98
PaO_2_/FiO_2_, mm Hg	EI	323 ± 110	272 ± 93	292 ± 97	296 ± 71	290 ± 87	281 ± 89	282 ± 110
	PCEA	336 ± 104	257 ± 83	300 ± 71	303 ± 103	298 ± 91	340 ± 129*	347 ± 124
	Control	1.00 ± 0.28	1.28 ± 0.63	1.50 ± 0.74^+^	1.75 ± 0.76^+^	1.74 ± 0.87^+^	1.80 ± 0.58^+^	1.50 ± 0.40^+^
Lactate, mmol/l	EI	1.07 ± 0.78	1.16 ± 0.36	1.38 ± 0.62	1.50 ± 0.74^+^	1.46 ± 0.81	1.44 ± 0.44	1.20 ± 0.37
	PCEA	1.06 ± 0.36	1.09 ± 0.42	1.40 ± 0.80^+^	1.81 ± 0.86^+^	1.70 ± 0.77^+^	1.35 ± 0.44^+^*	1.48 ± 0.71^+^
	Control	5.71 ± 1.51	6.77 ± 2.00^+^	8.01 ± 2.67^+^	8.42 ± 3.15^+^	7.22 ± 2.56^+^	6.17 ± 1.47	6.33 ± 1.36
Glucose, mmol/l	EI	5.16 ± 1.40	5.97 ± 1.44	7.28 ± 2.24^+^	6.90 ± 1.84^+^	6.25 ± 1.96^+^	5.64 ± 1.32	6.81 ± 2.34^+^
	PCEA	5.60 ± 1.48	6.41 ± 2.18^+^	7.68 ± 2.79^+^	7.84 ± 2.64^+^	7.12 ± 1.92^+^	6.13 ± 1.68^+^	6.59 ± 1.52
	Control	219 ± 80	-	-	984 ± 334^+^	-	714 ± 297^+^	-
Cortisol, nmol/l	EI	296 ± 111	-	-	861 ± 366^+^	-	618 ± 322^+^	-
	PCEA	303 ± 108	-	-	832 ± 371^+^	-	591 ± 223^+^	-
	Control	0.01 ± 0.00	-	-	0.32 ± 0.30^+^	-	0.25 ± 0.35	-
Troponin T, ng/ml	EI	0.01 ± 0.00	-	-	0.20 ± 0.11^+^	-	0.10 ± 0.08	-
	PCEA	0.01 ± 0.00	-	-	0.26 ± 0.20^+^	-	0.33 ± 0.52	-

Data are presented as mean ± SD.

*P < 0.05 compared with the control group;

^+ ^*P *< 0.05 compared with baseline (after induction of anesthesia).

Control, control group; EI, epidural infusion group; PCEA, patient controlled epidural analgesia group.

The VAS scores were within 20 mm at rest and 30 mm during coughing in all groups without intergroup differences excluding 12 h when VAS score was significantly lower in the PCEA group as compared to controls (Table 4). The level of postoperative sedation did not differ among the groups.

**Table 4:** Postoperative pain as assessed by visual analog scale (VAS) in patients undergoing OPCAB

Characteristic	Group	Time after surgery			
		6 hours	12 hours	18 hours	24 hours
	Control	20 (0-30)	20 (0-35)	20 (0-30)	10 (0-20)
VAS at rest	EI	15 (0-30)	10 (0-20)	10 (0-20)	0 (0-20)
	PCEA	0 (0-20)	0 (0-10)*	0 (0-18)	0 (0-18)
	Control	30 (0-55)	30 (20-50)	30 (20-50)	30 (20-50)
VAS during coughing	EI	20 (0-40)	30 (20-40)	30 (10-50)	30 (20-40)
	PCEA	20 (10-40)	20 (0-30)	30 (10-40)	20 (20-40)

Data are presented as median (25^th^-75^th ^percentiles). *P < 0.05 compared with the control group.

Control, control group; EI, epidural infusion group; PCEA, patient controlled epidural analgesia group.

Table 5 shows that during OPCAB, EA reduced the consumption of propofol by 15% and fentanyl by 50% (*P *< 0.05). The postoperative requirement of ropivacaine increased by 20% in the PCEA group as compared with the EI group (*P = *0.03). In both EA groups, the requirement of nitroglycerin decreased by a 7-fold intraoperatively and by a 2.5-fold after OPCAB (*P *< 0.05). Intraoperative inotropes/vasopressors were administered more frequently in the EA groups as compared with the control group (43% *vs*. 13%, respectively; *P *= 0.02). Colloids also were given more frequently in the patients receiving EA (75% *vs*. 37%, *P *= 0.01). After OPCAB, the incidence of colloid administration was higher in the EI group compared with the other groups (*P *< 0.05). Intraoperative fluid balance increased by 21% in the EA groups (*P *< 0.05). There were no significant differences in blood loss, urine output, administration of crystalloids and adverse events (not shown). There were no complications related to EA.

**Table 5:** Requirements of drugs and volume balance in patients undergoing OPCAB

Characteristic	Groups		
	Control (n = 30)	EI (n = 30)	PCEA (n = 30)
Propofol during surgery, mg	1200 (988-1400)	1000 (775-1100)*	1100 (900-1200)
Fentanyl during surgery, mg	1.2 (1.1-1.3)	0.6 (0.5-0.7)*	0.6 (0.5-0.9)*
Fentanyl during first 24 hrs after surgery, mg	0.70 (0.50-1.00)	0.27 (0.20-0.30)	0.30 (0.20-0.35)
Ropivacaine during surgery, mg	-	75 (75-100)	75 (75-100)
Ropivacaine during first 24 hrs after surgery, mg	-	250 (192-300)	300 (200-348)^#^
Nitroglycerin during surgery, mg	11.0 (6.7-19.5)	1.0 (0.0-2.3)*	2.2 (0.0-4.0)*
Nitroglycerin during first 24 hrs after surgery, mg	24.0 (16.0-33.5)	10.0 (0.0-15.0)*	9.0 (0.0-18.5)*
Inotropes/vasopressors during surgery, number of patients	5	15*	11
Inotropes/vasopressors during first 24 hrs after surgery, number of patients	0	5	1
Colloids during surgery, number of patients	11	21*	24*
Colloids during first 24 hrs after surgery, number of patients	7	16*	6^#^
Fluid balance during surgery, ml	1614 ± 537	1949 ± 475*	1919 ± 354

Data are presented as mean ± SD, median (25^th^-75^th ^percentiles), or number of patients.

* *P *< 0.05 compared with the control group;

^# ^*P *< 0.05 compared with the EI group.

Control: control group; EI, epidural infusion group; PCEA, patient controlled epidural analgesia group.

## Discussion

The present study demonstrates that EA with ropivacaine/fentanyl causes a moderate decrease in arterial pressure and prevents reduction of GEF and lung fluid accumulation during OPCAB. The epidural administration of ropivacaine and fentanyl reduces the requirement of nitroglycerin and intravenous agents for anesthesia and analgesia, but requires more frequent perioperative therapy with colloids and inotropes/vasopressors. Postoperatively, EI provides adequate analgesia and improves left ventricle myocardial contractility. Moreover, EI combined with PCA is associated with mild hyperventilation, transient improvement of oxygenation and tissue perfusion and decreased duration of mechanical ventilation after OPCAB in comparison with the control group.

The postoperative improvement of lung function observed in the PCEA group was accompanied by reduced time to tracheal extubation, but the durations of ICU and hospital stays did not differ significantly. These results are in accordance with other studies of EA in coronary surgery and can be explained by a wide range of confounding factors that are able to influence the length of hospitalization [8,10-17]. However, according to Sharma *et al.*, the use of epidural analgesia in obese patients can shorten the ICU stay after OPCAB [13]. This effect was explained by a reduced incidence of respiratory complications in this category of high-risk patients. Earlier hospital discharge after EA for CABG was also reported by de Vries *et al*. [18].

After induction of anesthesia, all the groups presented with myocardial dysfunction and systemic vasoconstriction, as judged by the occurrence of bradycardia, decreased CI, CFI and dPmax, and increased SVRI. The restraint of the heart was accompanied by a rise in CVP, paralleled by a decline in GEF and an increase in EVLW in the control group. This is typical for OPCAB and can be explained by "enucleation" of the heart, kinking of vessels, reduction of venous return, and impairment of ventricular geometry [1,19]. After OPCAB, we observed a reduction of systemic vascular tone and a rise in myocardial performance. These changes are consistent with other investigations of CABG and may result from the restoration of coronary blood flow and reversal of myocardial depression by goal-directed hemodynamic optimization [1,19,20]. As shown in several previous studies, volumetric parameters measured by transpulmonary thermodilution, such as GEDVI, which we used for hemodynamic optimization, is a more sensitive indicator of preload compared to CVP and can serve as guidance for colloid administration [19-24]. Most likely, this allowed us to maintain normal preload in all groups.

During OPCAB, EA decreased MAP transiently and partly prevented the decline in GEF as well as lung water accumulation, which we observed in the control group. In addition, in contrast to the control group, postoperative EI prompted a significant increase in dPmax. Although displaying only statistical intragroup differences, these changes can be explained by the hemodynamic effects of epidural blockade, including afterload reduction, that can lead to improvements in myocardial performance and pulmonary blood flow [7,10,25,26]. Recently, similar findings were noticed by investigators, who used EA in on-pump CABG [25,26].

The changes in hemodynamics observed during and after OPCAB were accompanied by transient metabolic acidosis and increased plasma lactate in parallel with hyperglycemia and rise in cortisol and troponin-T plasma concentrations in all groups. These data are consistent with results published by other authors and can be explained by tissue hypoperfusion, inflammation, surgical stress and myocardial damage caused by CABG [1,14,26,27].

We found that epidural analgesia after OPCAB resulted in mild hyperventilation. Moreover, the PCEA with ropivacaine/fentanyl led to transient postoperative improvement in arterial oxygenation and decreased lactate, possibly due to improvement of pulmonary and systemic perfusion [28]. In addition to these mechanisms, the advantageous respiratory effects of epidural blockade in cardiac surgery were associated with reduced incidence of postoperative atelectases and improved quality of analgesia [15-17,28]. In our study, epidural anesthesia and analgesia provided adequate pain control, similar to that observed after administration of opioids in the control group, as confirmed by VAS score < 30 mm in both epidural groups; optimal analgesia was observed after PCEA. Thus, the combined effects of analgesia, pulmonary vasodilation, prevention of lung edema and improvement of pulmonary mechanics might have resulted in a better lung function in the PCEA group that allowed earlier termination of respiratory support.

In the present study, the analgesic effect of epidural administration of ropivacaine and fentanyl reduced the requirements of intravenously administered fentanyl and propofol for general anesthesia. The postoperative use of PCEA led to increased consumption of ropivacaine but did not influence the incidence of adverse events after OPCAB, like oversedation, pruritus, nausea, vomiting or arrhythmias. This is consistent with other investigations in this field. By contrast, several authors report reduced incidence of atrial fibrillation after EA for coronary surgery, probably due to the sympatholythic action of epidural blockade [11,15,28-30]. Thus, by reducing the requirements in opioids, time to tracheal extubation and number of complications, EA can become part of a fast-track concept of cardiac anesthesia that is aimed to achieve cost-savings, and improve clinical outcome, as suggested by recent workers [31]. Despite several beneficial effects of EA, we found increased requirements for colloids and inotrope/vasopressor support to maintain targeted hemodynamic values. This led to increased intraoperative fluid balance that also might have influenced cardiopulmonary function. In parallel, thoracic epidural administration of ropivacaine and fentanyl resulted in significant reduction of perioperative nitroglycerin requirement. These changes can be explained by vasodilation and redistribution of blood volume caused by EA and analgesia. Thus the hypotensive effect of epidural blockade should not be underestimated, especially in hemodynamically unstable patients. Similar results were obtained by other authors studying EA in coronary surgery [10,11,28].

A limitation of this patient-controlled mode of analgesia is that its use depends on the condition of the patient. Moreover, some of the effects of PCEA on cardiopulmonary function occurred transiently and their clinical significances should be interpreted with caution. Therefore, larger studies are warranted to confirm our findings and to determine the optimal regimens of EA and postoperative analgesia in OPCAB patients.

## Conclusion

The use of EA during OPCAB reduces transiently arterial pressure and prevents lung fluid accumulation. Being a component of a goal-directed perioperative strategy, the epidural administration of ropivacaine/fentanyl can improve myocardial performance and provide analgesia comparable with intravenous opioids, although increasing the requirements for fluids and vasoactive therapies. After OPCAB, continuous EI combined with PCEA increases tissue perfusion and improves lung function, thus shortening the duration of mechanical ventilation.

## List of abbreviations

**CABG**: **c**oronary artery bypass grafting; **CI**: cardiac index; **CFI **-cardiac function index; **CO**: cardiac output; **CPB**: cardiopulmonary bypass; **CVP**: central venous pressure; **dPmax**: left ventricle contractility index; **EA**: epidural anesthesia; **EI**: epidural infusion; **ECG**: electrocardiogram; **EVLWI**: extravascular lung water index; **FiO_2_**: fraction of inspired oxygen; **GEDVI**: global end-diastolic volume index; **GEF**: global ejection fraction; **Hb**: blood hemoglobin concentration; **HR**: heart rate; **ICU**: intensive care unit; **IV**: intravenously; **MAP**: mean arterial pressure; **OPCAB**: off-pump coronary artery bypass grafting; **PaCO_2_**: partial pressure of carbon dioxide in arterial blood; **PaO_2_**: partial pressure of oxygen in arterial blood; **PCEA**: patient-controlled epidural analgesia; **SpO_2_**: peripheral oxygen saturation; **SVRI**: systemic vascular resistance index; **VAS**: visual analog scale.

## Competing interests

The authors declare that they have no competing interests.

## Authors' contributions

MYK, AVE and AAS participated in the design of the study, collected and analyzed the data, prepared figures and tables and drafted the manuscript. LJB participated in the design of the study and drafted the manuscript. All authors have read and approved the final manuscript.

## Pre-publication history

The pre-publication history for this paper can be accessed here:

http://www.biomedcentral.com/1471-2253/11/17/prepub
